# Dynamic Balance of Histone H3 Methylation: Regulatory Mechanisms and Therapeutic Prospects in RA Immune Dysregulation and Bone Metabolism Imbalance

**DOI:** 10.1155/jimr/5642138

**Published:** 2026-08-07

**Authors:** Shili Yang, Huaiquan Liu, Haiyang Kou, Lingyan Lai, Xinyan Zhang, Yunling Xu, Yu Sun, Bo Chen

**Affiliations:** ^1^ Guizhou University of Traditional Chinese Medicine, Guiyang 550025, Guizhou Province, China, gzu.edu.cn

**Keywords:** bone metabolism imbalance, histone H3 methylation, immune dysregulation, rheumatoid arthritis, therapeutic prospects

## Abstract

**Objective:**

To clarify the regulatory mechanisms of the dynamic balance of histone H3 methylation in rheumatoid arthritis (RA) immune dysregulation and bone metabolism imbalance, and to explore related therapeutic approaches.

**Methods:**

We systematically reviewed the roles of key histone H3 methylation sites, including H3K4me3, H3K9me3, and H3K27me3, in RA immune cell activation, inflammatory signaling pathway regulation, and bone cell differentiation. We analyzed the core functions of these sites in the cross‐regulation of “immune dysregulation—bone metabolism imbalance” and discussed therapeutic strategies targeting this methylation balance.

**Results:**

Accumulating preclinical evidence indicates that the dynamic balance of histone H3 methylation may regulate RA immune cell differentiation imbalance, excessive activation of inflammatory signaling pathways, and the imbalance state where bone resorption exceeds bone formation by modulating the activities of methyltransferases and demethylases. This balance appears to be a key mechanistic link connecting RA immune dysregulation and bone metabolism imbalance. Agents targeting this balance (such as EZH2 inhibitors and Janus kinase [JAK] inhibitors) have shown promising preclinical potential for dual anti‐inflammatory and bone‐protective effects.

**Conclusion:**

The dynamic balance of histone H3 methylation emerges as a potential core regulatory hub in the pathological process of RA based on preclinical studies, providing theoretical support for precise diagnosis and treatment of RA. Related targeting strategies may become a new direction to break through the limitations of existing treatments.

## 1. Introduction

Rheumatoid arthritis (RA) is an autoimmune multisystem chronic inflammatory disease with an unknown etiology [1]. It can affect people of all ages, with the 50–59 age group being the high‐incidence period. Globally, the prevalence of RA in adults is about 1/200, and the risk for women is 2–3 times higher than for men [2]. The disease process is closely related to synovial tissue hyperplasia, pannus formation, cartilage damage, and systemic complications [3]. It not only destroys joint structures but can also involve extra‐articular organs such as the heart, kidneys, lungs, digestive system, eyes, skin, and nervous system [4]. In severe cases, it may further induce disability, systemic complications, and even premature death [5, 6], causing heavy life, economic, and psychological burdens for patients and seriously interfering with their normal lives. Currently, commonly used clinical biological agents such as anti‐tumor necrosis factor‐α (TNF‐α) and anti‐interleukin‐6 (IL‐6) can alleviate inflammatory symptoms, but about 40% of patients have poor responses, and they have limited ability to fully reverse established bone tissue destruction [7, 8]. Therefore, it is still necessary to explore new therapeutic targets from the perspective of pathological mechanisms. Although significant progress has been made in the study of RA pathophysiological mechanisms, its exact etiology has not been fully elucidated [9]. It is generally believed that the progression of RA pathology highly depends on the synergistic imbalance of two core aspects: one is the chronic inflammatory amplification effect driven by immune dysregulation [9], and the other is the structural damage caused by bone metabolism imbalance [10]. At the immune level, T‐cell subset imbalance (such as increased Th17/Treg ratio [11]), macrophage activation, and abnormal proliferation of fibroblast‐like synoviocytes (FLSs) together build an inflammatory microenvironment and aggravate synovial inflammation by secreting cytokines such as TNF‐α, IL‐6, and IL‐17 [12–14]. At the bone metabolism level, excessive activation of osteoclasts and suppressed function of osteoblasts jointly lead to an imbalance state of “bone resorption > bone formation,” thereby causing bone erosion, periarticular bone loss, and systemic osteoporosis [15–18]. Recent preclinical studies have further demonstrated that “immune dysregulation—bone metabolism imbalance” in RA does not appear to occur independently but relies on the interaction between immune cells and stromal cells (such as FLS and osteoclasts) [19–21]. Moreover, the dynamic regulation of these cell heterogeneities and the continuous secretion of inflammatory factors appear to be regulated by epigenetic mechanisms [12, 22, 23]. In addition to histone methylation, peptidylarginine deiminase 4 (PAD4)‐mediated histone citrullination has emerged as another critical epigenetic modification closely linked to RA pathogenesis. PAD4 catalyzes the conversion of arginine residues to citrulline on histone tails, which can antagonize histone methylation at the same or adjacent sites and alter chromatin accessibility. Accumulating evidence indicates that aberrant PAD4 expression and activity contribute to immune cell activation, pro‐inflammatory cytokine production, and anticitrullinated protein antibody (ACPA) formation in RA, further exacerbating synovial inflammation and joint destruction [24]. Among them, histone H3 methylation modifications, because they can directly regulate the expression of inflammation‐ and bone metabolism‐related genes, have become key regulatory targets in this field [25–27]. However, current research mostly focuses on the independent roles of single H3 sites, and there is still a lack of systematic organization on the overall regulatory chain where changes in the balance of methyltransferases and demethylases induce abnormal H3 methylation states, leading to the synergistic imbalance of RA immune and bone metabolism [28]. Based on the above situation, this article takes “dynamic balance of histone H3 methylation” as the core theme, systematically reviews the mechanisms of action of three key sites—histone H3 lysine 4 trimethylation (H3K4me3), histone H3 lysine 9 trimethylation (H3K9me3), and histone H3 lysine 27 trimethylation (H3K27me3)—in RA immune cell activation, inflammatory signaling pathway regulation, and bone cell differentiation. It deeply analyzes the core functions of these sites in the cross‐regulation of “immune dysregulation—bone metabolism imbalance” and discusses RA treatment strategies targeting H3 methylation balance and their clinical translation potential in order to provide theoretical support and research directions for precise diagnosis and treatment of RA.

## 2. Search Strategy and Selection Criteria

### 2.1. Search Strategy

A comprehensive literature search was performed using PubMed as the primary database, supplemented by Web of Science, Scopus, and Google Scholar. The following keywords were combined for retrieval: “histone H3 methylation,” “rheumatoid arthritis,” “immune dysregulation,” “bone metabolism imbalance,” “epigenetic therapy,” “EZH2,” “H3K4me3”, and “H3K27me3.” The search covered all available records from database inception to May 2026, with 93.1% of the final included articles published between 2011 and 2026.

### 2.2. Inclusion Criteria

Articles were included if they met at least one of the following criteria: (i) original research directly investigating the role of histone H3 methylation in RA immune cell activation, inflammatory signaling regulation, or bone cell differentiation; (ii) systematic reviews comprehensively summarizing epigenetic regulatory mechanisms in RA; or (iii) classical basic studies elucidating the fundamental biological characteristics of histone methylation (e.g., enzymatic mechanisms and chromatin regulation), which provided the theoretical foundation for this review. Priority was given to original studies with rigorous experimental design, reproducible data, and both in vivo and in vitro validations.

### 2.3. Exclusion Criteria

The following types of literature were excluded: (i) irrelevant topics, including studies focusing only on other histone modifications without H3 methylation involvement, studies restricted to other autoimmune or bone/immune disorders unrelated to RA, and studies on other H3 sites lacking clear RA association; (ii) literature of insufficient quality, such as conference abstracts, theses, case reports, data‐free expert commentaries, duplicate publications, or studies with obvious methodological flaws (e.g., <3 in vitro replicates, <5 animals per group, lack of negative controls, and statistical errors); (iii) outdated, nonseminal studies published before 2010 without subsequent independent validation; and (iv) inaccessible articles lacking available full texts and sufficient abstract information.

### 2.4. Quality Control

For subfields with limited high‐quality original research (e.g., the role of H3K9me3 in RA FLS proliferation), authoritative review articles or methodologically sound original studies from mid‐tier journals were appropriately included to ensure the systematicity and completeness of this review. All 102 final included articles were independently reviewed and cross‐validated by at least two authors to confirm the accuracy of the cited content and its strict relevance to the study theme.

## 3. Basic Biological Characteristics of Histone H3 Methylation Dynamic Balance

### 3.1. Site Diversity and Functional Differences of Histone H3 Methylation

Histone H3 methylation modifications mainly occur on lysine and arginine residues. This process is catalyzed by methyltransferases (such as the SET family and PRMT family) [28, 29], while demethylases (such as the KDM family and LSD1) maintain dynamic balance through reverse regulation [30, 31]. These modifications are highly site‐specific, and the methylation state of different sites can precisely regulate gene expression by controlling chromatin compaction or loosening and transcription factor binding efficiency, exhibiting a core feature of “site determines function” [32, 33]. In the pathological processes of RA immune dysregulation and bone metabolism imbalance, the modification imbalance of core sites such as H3K4me3, H3K9me3, and H3K27me3 has been demonstrated in preclinical studies to play key regulatory roles. While the H3K4, H3K9, and H3K27 sites mediated by well‐known enzymes like MLL1 and EZH2 are deeply investigated, a deeper exploration of other modifications strengthens the comprehensiveness of the RA epigenetic landscape. For instance, H3K36me3 is generally linked to transcriptional elongation, and comprehensive epigenomic mapping of RA FLS has highlighted its crucial involvement in establishing the unique, aggressive epigenetic profile of these cells [27]. Similarly, H3R17me2 dynamically regulates specific inflammatory genes. Notably, H3 arginine methylation can be antagonized by PAD4‐mediated citrullination at the same or adjacent arginine residues. This dynamic competition between H3R17me2 and citrullination alters chromatin accessibility and contributes significantly to immune cell activation and synovial inflammation in RA [24, 34]. Expanding the focus beyond MLL1 and EZH2 to include other epigenetic modifiers (such as the SETD1A and KDM4 families) demonstrates that reshaping the chromatin landscape during immune responses is a highly coordinated, multitarget process [23, 35]. The core functional sites closely related to RA can be divided into activating and repressing modifications based on their regulatory effects on gene transcription. The imbalance between these two types is an important epigenetic basis for RA pathogenesis [36, 37]. Among them, the activating modification H3K4me3 is catalyzed by the histone H3K4 methyltransferase SET1/complex of proteins associated with Set1 (COMPASS) complexes and is widely recognized as a transcriptional activation marker. Traditionally, it is believed to be mainly enriched in gene promoter regions [38, 39], but recent studies indicate [32, 40] that a core role of H3K4me3 is to recruit the integrator complex subunit 11 (INTS11), releasing RNA polymerase II transcriptional pausing (pause‐release) and thereby promoting transcriptional elongation. In the RA pathological microenvironment, H3K4me3 modification patterns show significant abnormalities, which reflect a dysregulated balance driven by diverse epigenetic modifiers. In FLS, inflammatory factors like TNF‐α can activate MLL1, increasing H3K4me3 levels in the promoter regions of pro‐inflammatory genes such as IL‐6 and C–C motif ligand 2 (CCL2), ultimately forming a “cytokine‐epigenetic” positive feedback loop [25]. Additionally, the enrichment of the H3K4me3 activating mark in osteoblasts can directly enhance the transcription of the receptor activator of nuclear factor κB ligand (RANKL), promoting osteoclast differentiation and joint erosion. Notably, H3K4me3 levels in the promoter regions of IL‐6 and TNF‐α in circulating monocytes show no significant changes [41], indicating that local synovial microenvironment abnormalities in H3K4me3 represent one of the core mechanisms underlying RA immune‐bone metabolism cross‐dysregulation. H3K9me3 and H3K27me3 are both repressive modifications. H3K9me3, catalyzed by histone H3‐K9 methyltransferase 1 (SUV39H1), recruits heterochromatin protein 1 (HP1) to form dense constitutive heterochromatin structures, directly hindering transcription factor binding [42]. H3K27me3 is catalyzed by the Polycomb repressive complex 2 (PRC2) complex, which then recruits Polycomb repressive complex 1 (PRC1) to further catalyze histone H2A lysine 119 monoubiquitination (H2AK119ub), cooperatively maintaining facultative heterochromatin compaction and constructing a transcriptionally repressive microenvironment by binding inhibitors like enhancer of zeste homolog 2 (EZH2) [43, 44]. Both are enriched in the promoter regions of developmental genes in somatic cell nuclear transfer embryos, leading to significant chromatin compaction and gene silencing [44].

**Figure 1:**
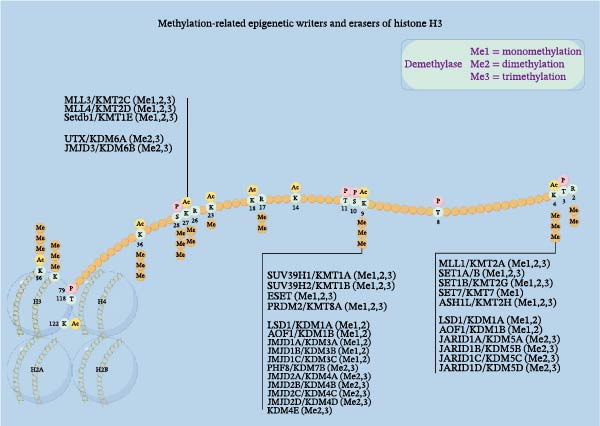
Schematic diagram of methylation‐related epigenetic writers and erasers targeting histone H3. The N‐terminal tail of histone H3 contains multiple highly conserved lysine (K) and arginine (R) residues that are subject to dynamic methylation. Specific methyltransferases (writers, e.g., MLL family, EZH2) and demethylases (erasers, e.g., JMJD3, KDM families) dynamically regulate the mono‐ (Me1), di‐ (Me2), and tri‐methylation (Me3) states at these specific sites. The dynamic balance of these modifications at core sites (such as H3K4, H3K9, and H3K27) strictly controls chromatin accessibility and plays a fundamental role in regulating target gene transcription.

The functional differences of the above core sites essentially depend on the dynamic regulation of specific methyltransferases (writers) and demethylases (erasers). The corresponding relationship between the sites and enzymes can be shown through histone H3 methylation modification, as illustrated in Figure 1.

### 3.2. Regulatory Mechanisms of Histone H3 Methylation

The dynamic balance of histone H3 methylation is key for cells to maintain normal epigenetic states and physiological functions. This balance relies on the precise coordination of the core molecular network of “writer‐eraser‐reader,” the dynamic integration of multilevel enzyme activity regulation mechanisms, and cross‐talk between epigenetic modifications. Any abnormality in these processes may lead to gene expression disorders and disease pathology [32, 45, 46]. Specifically, core regulatory factors form a closed‐loop system with strict site and functional specificity. Among the methyltransferases as “writers,” histone‐lysine N‐methyltransferase SETD1A (SET1A)/SET1B and the MLL family are core enzymes catalyzing H3K4me3 [47]. The myeloid/lymphoid or mixed‐lineage leukemia 3 complex (MLL3)/MLL4 complexes deposit H3K4me3 to regulate the sequential activation of development‐related genes [48, 49]. Among repressive modification‐related enzymes, the PRC2 core subunit EZH2 catalyzes H3K27me3 [50]. SUV39H1/2 and SET domain bifurcated histone lysine methyltransferase 1 (Setdb1) target H3K9me3 catalysis, maintaining heterochromatin and silencing regions like satellite DNA and endogenous retroviruses (ERVs) [51], suppressing transposon activation to avoid genomic disorders. The NSD family preferentially catalyzes H3K36me2, mainly acting in gene body regions, with catalytic activity crucial for transcription regulation and disease development [52]. Among the demethylases as “erasers,” the LSD family, represented by histone lysine‐specific demethylase 1A (KDM1A), relies on the flavin adenine dinucleotide (FAD) cofactor to catalyze H3K4me1/me2 demethylation [53]. The JmjC family includes most trimethylation‐specific demethylases [54], such as the lysine‐specific demethylase 5 (KDM5) family targeting H3K4me3 [55, 56]. KDM5B deficiency leads to failure in removing noncanonical H3K4me3 in early embryos, causing zygotic gene activation arrest [57]. The lysine demethylase 6 (KDM6) family specifically removes H3K27me3, with UTX (ubiquitously transcribed tetratricopeptide repeat, X chromosome) also serving as a component of the MLL3/MLL4 complex, achieving synergistic regulation of H3K27 demethylation and H3K4 methylation [58, 59]. “Reader proteins” containing domains like the chromodomain (e.g., HP1 family) recognize methylation sites to transmit regulatory signals, acting as key mediators converting methylation states into biological functions [60]. Additionally, enzyme activity is regulated at multiple levels by posttranslational modifications, metabolic states, and noncoding RNAs. At the posttranslational modification level, cyclin‐dependent kinase 1 (CDK1)/CDK2‐mediated EZH2 T350 phosphorylation enhances PRC2 recruitment efficiency, maintaining stable transmission of H3K27me3 marks [61]. CDK1‐mediated histone H3 threonine 3 phosphorylation (H3T3ph) synergizes with H3K4me3 by excluding the H3K4me3‐binding protein BPTF to promote chromatin remodeling, ensuring orderly chromatin state transitions during the cell cycle [62]. Ankyrin repeat and SOCS box containing 7 (ASB7), as a core component of the Cullin5 E3 ubiquitin ligase (CUL5‐E3), targets the degradation of the H3K9me3 methyltransferase SUV39H1 to maintain epigenetic homeostasis. During mitosis, CDK1 phosphorylation of ASB7 temporarily blocks its degradation function [42]. At the metabolic and signaling pathway level, the concentration of the methyl donor S‐adenosylmethionine (SAM) and the SAM/S‐adenosylhomocysteine (SAH) ratio directly affect the methyltransferase catalytic efficiency. An imbalance in this ratio can cause abnormalities in histone methylation like H3K27me3 [63]. Nicotinamide adenine dinucleotide (NAD+) can regulate sirtuin 1 (SIRT1) activity, mediating MLL1 deacetylation to inhibit its H3K4me3 methyltransferase function, thereby regulating circadian rhythm‐related gene epigenetic modifications [64]. The Wnt signaling pathway/β‐catenin pathway involves β‐catenin accumulation in the nucleus, recruiting histone coactivators. Meanwhile, EZH2 can bind to Wnt family gene promoters, suppressing their expression via H3K27me3, forming negative feedback regulation [65]. At the noncoding RNA level, the long noncoding RNA HOX transcript antisense RNA (HOTAIR) recruits EZH2 to promoter regions of target genes like E‐cadherin, inhibiting gene transcription through H3K27me3 mediation [66]. In summary, the regulation of histone H3 methylation dynamic balance is a complex network involving multiple factors and levels, with the activity balance of methyltransferases and demethylases as the core link. This balance is precisely regulated through posttranslational modifications, metabolic signals, and noncoding RNAs. An imbalance disrupts gene expression and drives disease, providing insights and theoretical support for RA‐related research.

## 4. Regulatory Mechanisms of the Dynamic Balance of Histone H3 Methylation in RA Immune Dysregulation

### 4.1. Regulation of Immune Cell Differentiation and Activation

The dynamic balance of key histone H3 methylation sites is a core regulatory mechanism for immune cell differentiation imbalance and abnormal activation in RA immune dysregulation. This balance dominates the pathological process by regulating the expression of immune cell‐specific genes [37, 67, 68]. In T‐cell subset differentiation, the increased Th17/Treg ratio is a well‐recognized core inducer of immune dysregulation in RA. Meng et al. [37] found that abnormal development of iNKT cells in the thymus of collagen‐induced arthritis mouse models is closely related to increased H3K4me3 and decreased H3K27me3 levels in key gene promoter regions. When MLL1 is activated by inflammatory factors like IL‐6 and transforming growth factor‐β (TGF‐β), it deposits H3K4me3 in the promoter region of the Th17 cell transcription factor retinoic acid receptor‐related orphan receptor C (RORC), promoting Th17 cell differentiation [69]. Zhao et al. [26] demonstrated that increased H3K27 demethylase activity in macrophages from RA patients and mouse models is associated with reduced H3K27me3 content in the promoter regions of RORC and IL‐6 genes. Meanwhile, the core transcription factor of Treg cells, Foxp3, shows transcriptional obstruction and functional weakening, which may be attributed to imbalanced H3K27me3 regulation and reduced H3K4me3 levels in its gene promoter region [37]. In macrophage polarization, Zhang et al. [12] indicated that targeting histone acetyltransferase K‐acetyltransferase 2A (KAT2A) in vitro can downregulate MLL1 activity, reducing H3K4me3 deposition in the promoter regions of M1‐type genes such as TNF‐α and IL‐1β, thereby inhibiting macrophage polarization toward a pro‐inflammatory phenotype. Tardito et al. [13] mentioned that H3K27me3 enrichment in the promoter regions of M2‐type genes (e.g., IL‐10, and Arg1) in RA patient macrophages is higher than in healthy individuals, suppressing macrophage conversion to an anti‐inflammatory phenotype. Okamoto et al. [25] also found that TNF‐α secreted by macrophages and CCL2 secreted by FLS are mediated at least in part by H3K4me3, further exacerbating pro‐inflammatory macrophage activation. In FLS activation, Ai et al. [27] comprehensive epigenetic mapping showed significantly increased H3K4me3 enrichment in the promoter regions of genes like IL‐6, CCL2, and matrix metalloproteinases (MMPs) in RA FLS, with “epigenetic memory” characteristics. Okamoto et al. [25] demonstrated that TNF‐α activates MLL1 via the mitogen‐activated protein kinase (MAPK)/extracellular regulated protein kinases (ERK) pathway in vitro, synergizing with reduced H3K27me3 levels in the IL‐6 gene promoter to promote sustained IL‐6 expression, forming a positive feedback loop maintaining FLS activation. Nygaard and Firestein [14] suggested that H3K9me3 may enrich in the p21 gene promoter via increased SUV39H1 activity, inhibiting p21 expression and releasing the suppression of FLS proliferation. Although this mechanism has not been directly verified in RA FLS, it has been confirmed in other inflammatory stromal cells [42].

### 4.2. Regulation of Immune Signaling Pathways

Histone H3 methylation has been shown to directly or indirectly regulate the activity of core immune signaling pathways in RA pathogenesis in preclinical studies by dynamically adjusting the methylation states of different sites, thereby reshaping immune cell functions and maintaining the inflammatory microenvironment homeostasis. Its regulatory roles mainly focus on classical inflammatory signaling pathways such as nuclear factor κB (NF‐κB), signal transducer and activator of transcription 3 (STAT‐3), and interferon (IFN), achieved through the synergistic action of specific methyltransferases and demethylases [36, 70–72]. In NF‐κB signaling regulation, as a core hub of RA inflammatory cascades, its activation process appears to be precisely and bidirectionally regulated by a diverse array of epigenetic modifiers [73]. For instance, the SET domain containing lysine methyltransferase 7 (SET7)/SET9 can catalyze H3K4 monomethylation to enhance NF‐κB p65 subunit binding efficiency to inflammatory gene promoters. Simultaneously, its methylation of nonhistone targets (e.g., RelA) may synergistically promote NF‐κB nuclear translocation and downstream pro‐inflammatory factor transcriptional activation [74]. Conversely, EZH2 can negatively regulate NF‐κB‐driven inflammation via H3K27me3 in certain cellular contexts. Preclinical studies indicate that EZH2 inhibits NLRP3 inflammasome activation, thereby reducing IL‐1β release. For instance, in inflammatory models such as ulcerative colitis, EZH2 accelerates NLRP3 degradation by regulating autophagy, which blocks the assembly of the NLRP3 complex and ultimately suppresses the NF‐κB pathway [75]. Although this mechanism has not been directly validated in RA, it suggests a potential conserved regulatory role of EZH2 in inflammatory diseases. In autoimmune inflammation, EZH2 can also use H3K27me3 to directly target and suppress the expression of the anti‐inflammatory gene suppressor of cytokine signaling 3 (Socs3) in macrophages, removing its promotion of TNF receptor‐associated factor 6 (TRAF6) ubiquitination degradation, thereby enhancing the NF‐κB pathway activity and pro‐inflammatory factor secretion [76]. Notably, EZH2 expression is indeed abnormally elevated in human RA synovial tissue samples, closely related to synovial cell inflammatory activation and NF‐κB pathway overactivation [77, 78]. Further in vitro studies show that silencing EZH2 was able to upregulate anti‐inflammatory molecules like Socs3, inhibit NF‐κB activity, and significantly reduce IL‐6 and IL‐1β secretion levels in macrophages, directly supporting its regulatory role in inflammatory pathways [76]. Additionally, the H3K4 methyltransferase Ash1l can induce the expression of inhibitory factor A20 through its methyltransferase activity. Via A20’s deubiquitination of inflammatory signaling molecules NEMO and TRAF6, it suppresses downstream NF‐κB inflammatory signaling and IL‐6 expression, preventing inflammatory autoimmune diseases [79]. In STAT‐3 signaling regulation, this pathway plays a key role in RA synovial fibroblast (FLS) activation and matrix‐degrading enzyme synthesis, with functions closely related to H3K4me3 activating modifications. Studies have found [36] that in RA patient FLS, H3K4me3 levels are significantly increased in the promoter regions of MMP genes like MMP‐1, MMP‐3, and MMP‐13, while H3K27me3 levels are decreased, forming a chromatin state conducive to transcription. Further mechanism verification [36] shows that WD repeat domain 5 (WDR5), a core component of the H3K4 methyltransferase complex, has upregulated expression that enhances STAT‐3 binding ability to MMP gene promoters. In vitro studies using human RA synovial fibroblasts demonstrated that silencing WDR5 reduced H3K4me3 levels and effectively inhibited IL‐6‐induced MMP expression. This regulatory network not only explains the molecular basis of RA joint destruction but also reveals the synergistic pathogenic role of histone methylation and cytokine signaling pathways. In IFN signaling regulation, abnormal activation of this pathway is key for establishing inflammatory phenotypes in RA monocytes, with activity regulated epigenetically by H3K27me3 modifications. Studies on juvenile idiopathic arthritis (JIA, a model for inflammatory arthritis) patient joint fluid monocytes show [80] that upregulated expression of IFN‐related genes (e.g., STAT1) is accompanied by reduced H3K27me3 modifications in promoter regions and increased chromatin openness. The clinically approved Janus kinase (JAK) inhibitor ruxolitinib has been shown to reverse abnormal H3K27me3 distribution in monocytes in ex vivo patient sample models by inhibiting IFN signal transduction, reshaping the enhancer landscape, and downregulating disease‐related gene expression, ultimately suppressing inflammatory phenotypes. This suggests cross‐regulation between histone H3 methylation and JAK‐STAT pathways, providing new ideas and directions for RA epigenetic regulation therapy.

## 5. Regulatory Mechanisms of Histone H3 Methylation Dynamic Balance in RA Bone Metabolism Imbalance

### 5.1. Regulation of Osteoclast Differentiation and Bone Resorption

Osteoclasts are currently recognized as the only multinucleated cells responsible for bone resorption in bone metabolism. Their differentiation, maturation, and functional activation rely on precise regulation of signaling pathways like RANKL–RANK‐NF‐κB. The dynamic balance of histone H3 methylation, by reshaping the chromatin structure and regulating target gene transcription, has emerged as a key epigenetic mechanism in this process based on preclinical studies. Its imbalance directly drives abnormal bone resorption and skeletal diseases. During osteoclast differentiation, H3K27 methylation shows dynamic regulation characteristics. EZH2, as the core methyltransferase of the Polycomb repressive complex PRC2, has been shown to directly silence the transcription of osteoclast differentiation negative regulators like MafB and Irf8 by catalyzing H3K27me3 modifications in preclinical models. Simultaneously, EZH2 can also interact with AKT in the cytoplasm through nonepigenetic mechanisms, promoting RANKL‐induced AKT phosphorylation and activating the mTOR signaling pathway, forming a dual regulatory mechanism involving both nuclear epigenetic regulation and cytoplasmic signal activation [81]. Additionally, the EZH2 selective inhibitor GSK126 has been shown to significantly disrupt osteoclast actin ring structures and reduce bone resorption activity in in vitro osteoclast differentiation assays and in vivo CIA mouse models [81]. Jumonji domain containing 3 (JMJD3) activates the transcription of nuclear factor of activated T‐cells, cytoplasmic 1 (NFATc1) by removing H3K27me3 modifications in its gene promoter region. Silencing the JMJD3 gene blocks this process and inhibits osteoclastogenesis [82]. Additional sex combs‐like 1 (ASXL1) typically recruits the PRC2 to maintain suppressive H3K27me3 marks on osteoclastogenic genes like NFATc1. However, ASXL1 deficiency disrupts this epigenetic balance, causing a decrease in H3K27me3 and a specific increase in activating H3K4me3 marks. Furthermore, this deficiency upregulates the demethylase JMJD3, strongly enhancing NFATc1 transcription and thereby exacerbating osteoclastogenesis and bone resorption [83]. Moreover, H3K27me1 modifications can directly bind to the zinc finger domain of MMP‐9, providing precise recognition sites for MMP‐9 to target the promoter regions of osteoclast differentiation factor genes (e.g., NFATc1 and CTSK). MMP‐9 specifically cleaves downstream of histone H3‐K27 monomethylation (K27me1) in the histone H3 N‐terminal tail (H3NT), disrupting nucleosome stability and exposing transcription factor binding sites like activator protein 1 (AP‐1), ultimately activating osteoclast‐specific gene expression networks to promote osteoclastogenesis [84]. In H3K9 methylation regulation, in mechanically unloaded‐induced bone loss models, the histone methyltransferase G9a expression is upregulated, increasing H3K9me2/me3 levels in the lncRNA H19 promoter region. By forming a complex with DNA methyltransferase 1 (DNMT1) and ubiquitin‐like with PHD and ring finger domains 1 (UHRF1), it synergistically suppresses lncH19 expression and osteogenic activity. Knocking down G9a in these mechanically unloaded mouse models significantly alleviated bone loss [85]. Furthermore, G9a regulates cell activity through different methylation modifications in osteoclast precursors and osteoblasts, respectively, and this dual role exacerbates bone metabolism imbalances [86]. H3K4me3, as a transcriptional activation mark, regulates key osteoclast differentiation genes through dynamic switching with H3K27me3. For example, the protocadherin 7 (PCDH7) gene exhibits a bivalent state in osteoclast precursors and switches to an H3K4me3+ monovalent state with high expression after RANKL stimulation, promoting cell fusion‐related gene expression and accelerating osteoclast multinucleation [87]. Cell adhesion molecule 1 (Cadm1), as a direct target gene of NFATc1, is highly expressed after differentiation and maturation, forming a negative feedback loop by inhibiting osteoclast survival and fusion activity to prevent excessive bone resorption activation [88]. In RA bone destruction, EZH2 expression is significantly upregulated in joint tissues of collagen‐induced arthritis mouse models and positively correlated with arthritis indices. EZH2 may recruit the PRC2 to catalyze H3K27me3 modifications in the promoter region of the osteogenesis‐related gene N‐myc downstream‐regulated gene 2 (NDRG2), thereby inhibiting NDRG2 transcription. This regulation further leads to a bidirectional imbalance of suppressed osteogenic differentiation and enhanced osteoclastogenesis, ultimately aggravating joint bone destruction [89]. Knocking down EZH2 in collagen‐induced arthritis mice significantly reduced arthritis indices, alleviated joint inflammation and bone destruction, decreased tartrate‐resistant acid phosphatase‐positive osteoclast numbers, and increased bone density [89]. Epigenetic regulation in RA joint destruction can also occur through abnormal synovial cell activation. For example, JMJD3 is highly expressed in RA patient synovial fibroblasts, promoting synovial hyperplasia via H3K27 demethylation of proliferating cell nuclear antigen (PCNA), indirectly exacerbating bone erosion [90].

### 5.2. Regulation of Osteoblast Differentiation and Bone Formation

The dynamic balance of histone H3 methylation has been shown to profoundly affect osteoblast differentiation and bone formation processes in preclinical studies by regulating key transcription factor activity and chromatin states. The H3K27me3 demethylase Utx is upregulated during osteoblast differentiation, promoting osteoblast differentiation by regulating the H3K27me3 dynamic balance. Its deficiency significantly weakens alkaline phosphatase (ALP) activity and calcium deposition capacity [91]. Similarly, the H3K27 demethylase Jmjd3 promotes osteoblast differentiation by relieving H3K27me3 repression of runt‐related transcription factor 2 (Runx2) and osterix (OSX), upregulating osteogenic markers like osteopontin and bone sialoprotein [92]. In H3K9 methylation, the methyltransferase ESET regulates mesenchymal stem cell differentiation into osteoblasts, and its deficiency leads to impaired bone formation [93]. H3K4me3, as an activating histone modification, is maintained by the MLL/COMPASS complex, which enriches in the Runx2‐P1 promoter region to promote Runx2 transcription via H3K4me3 enrichment [94]. The lysine‐specific demethylase 5A (KDM5A) negatively regulates mesenchymal stem cell osteogenic differentiation by reducing H3K4me3 levels in the promoter regions of genes like Runx2 and osteocalcin. Its high expression in steroid‐induced osteonecrosis is an important mechanism for impaired bone formation [95]. Together, the synergistic actions of EZH2, UTX, JMJD3, ESET, and KDM5A demonstrate that osteoblast differentiation is governed by a highly complex, multienzyme epigenetic network rather than a single regulatory axis.

## 6. Cross‐Regulation of “Immune‐Bone Metabolism” by Histone H3 Methylation Dynamic Balance in RA

In RA, the dynamic balance of histone H3 methylation appears to act as a core epigenetic hub connecting immune inflammation and bone metabolism disorders. Rather than acting in isolation, these epigenetic modifications create a complex cross‐talk network between immune cells and stromal cells. For example, in RA synovial fibroblasts, IL‐6 synergizes with altered methylation states (increased H3K4me3 and decreased H3K27me3) to promote the expression of matrix‐degrading enzymes, directly exacerbating joint destruction [36]. Additionally, although the direct H3 methylation mechanisms in RA Th17 cell differentiation require further validation, the H3K27me3/JMJD3/EZH2 axis has been shown to regulate Th17 differentiation in related rheumatic diseases, suggesting a shared epigenetic pathway in immune dysregulation [96].

Instead of viewing these pathways separately, recent preclinical evidence highlights the synergistic and dual pathological roles of specific epigenetic enzymes. For instance, JMJD3 not only activates osteoclast differentiation by removing H3K27me3 marks on the NFATc1 promoter [82] but also paradoxically regulates osteoblast differentiation [92] and apoptosis [97]. Similarly, EZH2 concurrently exacerbates synovial inflammation by participating in FLS proliferation and invasion [77] and promotes bone resorption via nuclear and cytoplasmic mechanisms [81].

Because these epigenetic enzymes may simultaneously drive inflammatory amplification and bone erosion, targeted interventions hold the potential for dual therapeutic benefits. For example, the EZH2 inhibitor GSK126 has been shown to simultaneously inhibit RA inflammation and bone erosion in preclinical models [81]. Other cross‐talk mechanisms, such as the synergy between histone and DNA methylation mediated by enzymes like G9a [85], and the induction of osteoclast formation by carbamylated histones [98], further complete this regulatory network. This highlights that the RA epigenetic landscape is built upon a broad arsenal of modifiers acting in concert rather than relying solely on heavily studied targets like MLL1 and EZH2.

**Figure 2:**
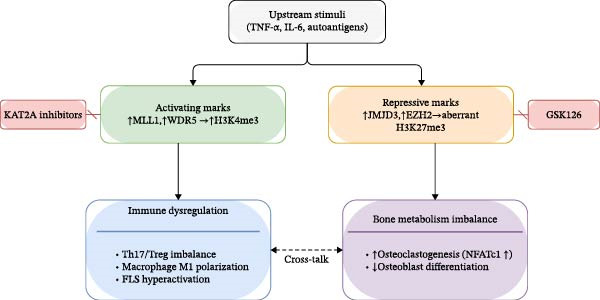
Upstream‐to‐downstream flow diagram of RA disease progression driven by histone H3 methylation and potential targeted interventions. Upstream inflammatory stimuli trigger aberrant expression of epigenetic writers (e.g., MLL1 and EZH2) and erasers (e.g., JMJD3). These epigenetic alterations drive downstream immune dysregulation (e.g., Th17/Treg imbalance, FLS activation) and bone metabolism imbalance (e.g., enhanced osteoclastogenesis). Pharmacological drug targets (e.g., GSK126, KAT2A inhibitors) can arrest disease progression by blocking specific epigenetic enzymes.

To visually summarize these complex pathogenic mechanisms and therapeutic strategies, Figure 2 provides an upstream‐to‐downstream flow diagram illustrating how histone H3 methylation drives RA progression and highlights possible drug targets. Additionally, the specific regulatory roles are detailed in Table 1, whereas potential therapeutic agents targeting histone H3 methylation, together with their epigenetic mechanisms, major biological effects, and supporting study models, are summarized in Table 2.

**Table 1:** Preclinical and clinical evidence for the regulation of immune dysregulation and bone metabolism imbalance in RA by histone H3 methylation homeostasis.

Regulatory targets	Key methylation sites and modification types	Regulatory mechanisms
Rheumatoid arthritis (RA)‐related immune dysregulation	H3K4me3 (activating modification)	1. Activates mixed‐lineage leukemia 1 (MLL1) to promote the expression of retinoic acid receptor‐related orphan receptor C (RORC), a transcription factor of Th17 cells, thereby driving Th17 cell differentiation. 2. Increases the expression of pro‐inflammatory genes such as interleukin‐6 (IL‐6) and C–C motif ligand 2 (CCL2) in fibroblast‐like synoviocytes (FLS), forming a “cytokine‐epigenetics” positive feedback loop. 3. Promotes the polarization of macrophages toward the pro‐inflammatory phenotype.
	H3K27me3 (repressive modification)	1. Enhanced activity of demethylases leads to reduced modification in the promoter regions of RORC and IL‐6 genes, resulting in increased secretion of pro‐inflammatory factors. 2. Dysregulated modification in the promoter region of forkhead box P3 (Foxp3), a core transcription factor of Treg cells, combined with decreased H3K4me3 levels, impairs transcription and weakens cell function.
	H3K9me3 (repressive modification)	May increase the enrichment of H3K9me3 in the promoter region of the p21 gene by upregulating the activity of suppressor of variegation 3‐9 homolog 1 (SUV39H1), thereby inhibiting p21 expression and relieving the suppression of FLS proliferation^a^.
RA‐related bone metabolism imbalance	H3K4me3 (activating modification)	1. Enhances the transcription of receptor activator of nuclear factor κB ligand (RANKL) in osteoblasts, promoting osteoclast differentiation. 2. Activates the protocadherin 7 (PCDH7) gene in osteoclast precursors to accelerate osteoclast multinucleation.
	H3K27me3 (repressive modification)	1. Osteoclasts: enhancer of zeste homolog 2 (EZH2) catalyzes H3K27me3 modification to silence negative regulators of osteoclast differentiation, such as MAF BZIP transcription factor (MafB) and interferon regulatory factor 8 (Irf8); jumonji domain containing 3 (JMJD3) mediates demethylation to activate nuclear factor of activated T‐cells, cytoplasmic 1 (NFATc1), promoting osteoclast formation. 2. Osteoblasts: Ubiquitously transcribed tetratricopeptide repeat, X chromosome (UTX) and JMJD3 mediate demethylation to relieve the suppression of runt‐related transcription factor 2 (Runx2) and osterix (OSX), facilitating osteoblast differentiation.
	H3K9me3 (repressive modification)	1. Osteoclasts: G9a methyltransferase (G9a) catalyzes H3K9me3 modification to inhibit the expression of long noncoding RNA H19 (lncH19), exacerbating bone loss. 2. Osteoblasts: SET domain bifurcated histone lysine methyltransferase 1 (ESET) regulates the differentiation of mesenchymal stem cells into osteoblasts, and its deficiency leads to impaired bone formation.

*Note:* Most mechanistic interactions described in this table are derived from in vitro cell culture experiments and in vivo animal model studies. Only a small number of findings have been validated in human RA patient tissue samples.

^a^The specific mechanism by which H3K9me3 relieves the suppression of cell proliferation via p21 has currently been robustly validated in other inflammatory stromal cells; its direct confirmation within RA‐specific FLS warrants further investigation.

**Table 2:** Potential therapeutic agents targeting histone H3 methylation for RA.

Targeting enzyme/pathway	Agent/drug	Epigenetic mechanism	Major biological effects	Study model
EZH2 (H3K27me3 writer)	GSK126	Decreases H3K27me3 levels; alters expression of negative regulators (e.g., MafB)	Reduces osteoclast actin ring formation; downregulates FLS matrix‐degrading enzymes	In vitro/CIA mouse model
EZH2 (H3K27me3 writer)	siRNA/shRNA	Silences EZH2 gene expression; upregulates Socs3	Inhibits NF‐κB activity; reduces IL‐6 and IL‐1β secretion	In vitro macrophages
JAK/STAT pathway	Ruxolitinib	Inhibits IFN signal transduction; reverses abnormal H3K27me3 distribution	Downregulates disease‐related gene expression; suppresses inflammatory phenotype	Ex vivo JIA patient monocytes
KAT2A/MLL1	KAT2A inhibitors	Downregulates MLL1 activity; reduces H3K4me3 deposition	Inhibits macrophage polarization toward M1 pro‐inflammatory phenotype	In vitro cell models

*Note:* The therapeutic strategies summarized in this table are primarily supported by preclinical studies, including in vitro cellular experiments, animal models, and ex vivo patient‐derived samples. Their specific efficacy and safety profiles for RA treatment require further clinical investigation.

## 7. Discussion

The dynamic balance of histone H3 methylation is increasingly recognized as a core component of the epigenetic regulatory network and has emerged as a key regulatory hub for the synergistic occurrence of “immune dysregulation—bone metabolism imbalance” in RA based on accumulating preclinical evidence. Mechanistically, the three core sites, H3K4me3, H3K9me3, and H3K27me3, exert critical regulatory effects on immune cell differentiation, inflammatory signaling pathways, and bone cell functions through dynamic switching of “activation‐repression” modifications. This regulation does not exist independently but forms a cross‐cell type regulatory network through the functional balance of methyltransferases and demethylases. For example, EZH2, as the core methyltransferase of H3K27me3, can promote bone resorption through dual mechanisms, silencing osteoclast negative regulators MafB and Irf8 in the nucleus [81] and activating the AKT‐mTOR pathway in the cytoplasm. Simultaneously, EZH2 is highly expressed in human RA synovial fibroblast samples, exacerbating joint destruction by regulating matrix‐degrading enzyme gene expression [77], further supporting the potential core role of the H3 methylation balance in linking immune inflammation and bone metabolism disorders. Additionally, JMJD3‐mediated H3K27me3 demethylation not only activates the key osteoclast differentiation gene NFATc1 [82] but also promotes osteoblast differentiation by relieving repression of Runx2 and OSX [92], indicating that a single demethylase can target different cell types to participate in regulating the “resorption‐formation” balance of bone metabolism. The imbalance of this role is an important epigenetic basis for RA bone destruction. Unlike previous studies focusing on the independent functions of single H3 methylation sites, this review systematically synthesizes the dynamic balance network of methyltransferases and demethylases, further revealing the regulatory chain of “enzyme activity abnormalities inducing H3 methylation state imbalance, thereby causing gene expression disorders, and ultimately driving RA pathological progression.” This systematic perspective provides a new framework for understanding the complex pathological mechanisms of RA. For example, at the immune dysregulation level, MLL1, as a key methyltransferase of H3K4me3, after activation by inflammatory factors like TNF‐α and IL‐6, not only promotes the expression of pro‐inflammatory genes like IL‐6 and CCL2 in FLS [25] but also promotes Th17 cell differentiation through H3K4me3 enrichment in the RORC promoter region [37], forming a positive feedback loop of “inflammatory factors–epigenetic modifications–immune activation.” Treg cell function inhibition is directly related to increased H3K27me3 and decreased H3K4me3 levels in the Foxp3 promoter region [37]. This cell‐specific difference in H3 methylation modifications provides epigenetic evidence for explaining the molecular mechanism of Th17/Treg imbalance in RA. At the bone metabolism level, ASXL1 maintains H3K27me3 levels by recruiting the PRC2. Its deficiency leads to reduced H3K27me3 and increased H3K4me3 in osteoclast key gene promoters, accompanied by upregulated JMJD3 expression, further exacerbating the H3 methylation imbalance and bone resorption [83]. This finding suggests that synergistic dysregulation of methyltransferases and demethylases may be an important inducement for RA bone metabolism imbalance, whereas previous studies mostly separately discussed the roles of certain enzymes without fully considering their balance relationship.

From a clinical translation perspective, regulatory factors related to the dynamic balance of histone H3 methylation provide new therapeutic targets with both anti‐inflammatory and bone‐protective potential for RA treatment, which are expected to break through the limitations of existing treatment methods. Current commonly used clinical anti‐TNF‐α and anti‐IL‐6 biological agents can alleviate inflammatory symptoms, but about 40% of patients have poor responses and cannot reverse existing bone destruction [7, 8]. Strategies targeting the H3 methylation balance can simultaneously intervene in immune inflammation and bone metabolism disorders by regulating epigenetic states. For example, the EZH2 inhibitor GSK126 not only disrupts osteoclast actin ring structures and reduces bone resorption activity [81] but also alleviates joint inflammation by downregulating matrix‐degrading enzyme expression in FLS [89] and has been confirmed in CIA models to reduce arthritis indices and increase bone density [89]. Additionally, the clinically approved JAK inhibitor ruxolitinib has been shown to reverse abnormal H3K27me3 distribution in monocytes in ex vivo patient sample models by inhibiting IFN signal transduction and downregulating IFN‐related gene expression [80], suggesting that the combined use of epigenetic modification inhibitors and signaling pathway inhibitors may produce synergistic therapeutic effects. Meanwhile, the H3K4me3 demethylase KDM5A is highly expressed in steroid‐induced osteonecrosis, inhibiting osteogenic differentiation by reducing H3K4me3 levels in the Runx2 promoter region [95]. This mechanism provides a potential target for RA complicated with osteoporosis, indicating that targeting the H3 methylation balance may achieve the dual therapeutic goal of “anti‐inflammatory‐bone protection.” Although accumulating preclinical research has indicated a key role of H3 methylation dynamic balance in RA pathology, several critical issues remain unresolved. First, current studies are predominantly based on cell lines or animal models, and the H3 methylation heterogeneity of different cell types in the human RA synovial tissue has not been clearly explained. Second, the cross‐talk mechanisms between histone methylation and other epigenetic or metabolic modifications (e.g., DNA methylation, noncoding RNA regulation, or metabolic factors like SAM and NAD+) require further elucidation.

Beyond these basic research limitations, translating these findings into RA clinical applications faces significant hurdles. Currently, detailed coverage of ongoing clinical trials reveals a striking gap: direct inhibitors of histone methylation enzymes (such as EZH2 inhibitors) are predominantly evaluated in oncological trials rather than in autoimmune diseases. For example, the EZH2 inhibitor tazemetostat has received FDA approval for specific sarcomas and lymphomas, demonstrating an acceptable safety profile in cancer patients [99]. However, clinical trials specifically repurposing these direct epigenetic drugs for RA are still in their infancy. The primary translational challenge lies in the ubiquitous nature of epigenetic enzymes. Because H3 methylation is fundamental to basic cellular functions and chromatin stability across all tissues, systemic administration of these inhibitors carries a potential high risk of severe off‐target toxicity and broad immunosuppression [100]. Furthermore, unlike cancer therapy, where the goal is often the eradication of malignant cells, RA therapy requires a delicate “epigenetic resetting” to restore immune homeostasis without abolishing normal immune defenses [101]. While indirect epigenetic modulation is already occurring in the clinic—such as with FDA‐approved JAK inhibitors that consequentially alter H3K27me3 landscapes—the development of RA‐specific, highly selective direct epigenetic agents remains a critical translational bottleneck [14, 80].

Taken together, the synthesis of available evidence presented in this review indicates that the future development of RA therapeutics may increasingly shift from broad immunosuppression towards more targeted epigenetic reprogramming. To overcome the aforementioned challenges and basic research limitations, future research could usefully explore three promising directions. First, the development of cell‐specific targeted delivery systems would be highly valuable. Utilizing nanomedicine or antibody‐drug conjugates to deliver epigenetic inhibitors selectively to hyperactive macrophages or aggressive FLSs could potentially maximize local efficacy while minimizing systemic risks. Second, advancing targeted protein degradation technologies, such as PROTACs, might offer a more durable therapeutic effect by mediating the selective degradation of disease‐driving epigenetic enzymes rather than merely blocking their catalytic domains. Third, establishing comprehensive H3 methylation signatures from the patient synovial fluid via single‐cell epigenomics could fundamentally transform patient stratification. Importantly, expanding these biomarker panels beyond the canonical H3K4/K9/K27 marks to include functionally diverse modifications (such as H3K36me3 for aggressive FLS phenotypes and H3R17me2 for specific inflammatory states) could precisely identify which patients are most likely to benefit from specific inhibitors, thereby facilitating personalized combination therapies. For instance, integrating epigenetic modulators with emerging receptor‐targeted agents—such as inhibitors targeting discoidin domain receptor 2 (DDR2), which has shown promising therapeutic potential against cartilage degeneration in joint microenvironments [102]—could provide synergistic protection against structural joint destruction. Fourth, exploring the cross‐talk between histone H3 methylation and other epigenetic modifications (such as PAD4‐mediated citrullination) and understudied H3 marks (including H3K36me3 and H3R17me2) will provide a more comprehensive understanding of RA epigenetic pathogenesis.

In conclusion, restoring the dynamic balance of histone H3 methylation might offer a unique opportunity to address the two most destructive aspects of RA simultaneously, namely, immune‐driven inflammation and structural bone erosion. Developing precision epigenetic therapies based on this dual‐efficacy mechanism is widely considered a promising and transformative frontier for the future management of RA.

## Author Contributions

Conceptualization: Shili Yang and Bo Chen. Data curation, formal analysis, software, visualization, writing – original draft: Shili Yang. Funding acquisition, project administration, supervision: Bo Chen. Methodology: Xinyan Zhang and Haiyang Kou. Resources: Yu Sun, Yunling Xu, and Xinyan Zhang. Investigation: Lingyan Lai and Yu Sun. Writing – review and editing: Bo Chen, Huaiquan Liu.

## Funding

This project was supported by the National Natural Science Foundation of China (Grant 82360976) and the Graduate Research Fund Project of Guizhou University of Traditional Chinese Medicine (Grant YCXKYB2026002).

## Disclosure

All authors agreed that this study was published in the *Journal of Inflammation*.

## Ethics Statement

This review did not require ethical approval.

## Conflicts of Interest

The authors declare no conflicts of interest.

## Data Availability

Data sharing is not applicable to this article as no datasets were generated or analyzed during the current study.
